# The learning-relative hemodynamic modulation of cortical plasticity induced by a force-control motor training

**DOI:** 10.3389/fnins.2022.922725

**Published:** 2022-09-08

**Authors:** Yongrong Wang, Shuai Feng, Rui Yang, Wensheng Hou, Xiaoying Wu, Lin Chen

**Affiliations:** ^1^Key Laboratory of Biorheological Science and Technology of Ministry of Education, Chongqing University, Chongqing, China; ^2^Chongqing Key Laboratory of Artificial Intelligence and Service Robot Control Technology, Chongqing University, Chongqing, China; ^3^Collaborative Innovation Center for Brain Science, Chongqing University, Chongqing, China; ^4^Chongqing Medical Electronics Engineering Technology Research Center, Chongqing University, Chongqing, China

**Keywords:** motor learning, hemodynamic modulation, plasticity, functional near infrared spectroscopy (fNIRS), motor cortex

## Abstract

**Background:**

Novel motor skills are generally acquired through repetitive practices which are believed to be strongly related to neural plasticity mechanisms. This study aimed to investigate the learning-relative hemodynamic modulation of cortical plasticity induced by long-term motor training.

**Methods:**

An 8-day participation-control program was conducted. Eighteen right-handed healthy participants were recruited and randomly assigned into the training (12) and control groups (6). The training group were arranged to undergo the 8-day block-designed motor training which required to repeat a visuomotor force-control task. The functional near-infrared spectroscopy (fNIRS) was used to continuously monitor the cortical hemodynamic response during training. Two transcranial magnetic stimulation (TMS) measurements were performed before and after training to evaluate the cortical excitability changes. The transfer effects of learning were also investigated.

**Results:**

The behavior performance was quantified *via* score execution accuracy to illustrate the fast/slow learning stages as experience cumulated. The cortical hemodynamic activations mapped by fNIRS exhibited a temporal evolution trends that agreed the expansion–renormalization model, which assumed the brain modulation against skill acquisition includes complex mechanisms of neural expansion, selection, and renormalization. Functional connectivity (FC) analysis showed the FC strength was maintained, while the measured homodynamic activation returned to baseline after certain level of skill acquisition. Furthermore, the TMS results demonstrated a significant increase of motor evoked potential (MEP) on the targeted muscle for the trained participants, who significantly outperformed the untrained subjects in learning transfer investigation.

**Conclusion:**

The study illustrated the expansion–renormalization trends during continuous motor training, and relative analysis showed the functional connectivity enhancement may be maintained after amplitude renormalization of cortical hemodynamic activations. The TMS findings further gave an implication of neural facilitations on the descending motor pathway when brain activation returned to renormalization status after certain level of learning stages was achieved, and the learning can transfer to enhance the performance while encountering similar tasks.

## Introduction

Motor behaviors play a fundamental role in human daily lives. It is well-acknowledged that motor skills are generally acquired and retained with accuracy improvements through repetitive practices (Willingham, 1998), during which the brain inherently adapts itself both functionally and structurally to the changing circumstances and allows the relative neural circuitry to establish a robust optimal pattern in response to experience (Floyer-Lea and Matthews, 2005; Xu et al., 2009; Yang et al., 2009; Wenger et al., 2017). In neuroscience, this capability of human brain is referred to as neural plasticity that enables the nervous system modification to various complicating or changing environments for new behavior learning (Cooke and Bliss, 2006; Dayan and Cohen, 2011; Makino et al., 2016). The ability of plasticity peaks at a critical period in childhood; nevertheless, novel findings have collected sufficient evidence that the adult cortex maintained this flexibility for reorganization throughout the lifespan (Sanes and Donoghue, 2000; Lövdén et al., 2010). The plasticity in adult brain not only ensures further skill acquisitions in face of changing mental or behavior demands, but also highlights the possibility of intervention for function recovery after brain injury such as stroke (Maddalena et al., 2015; Lotze et al., 2019). Thus, plasticity makes the brain to be flexible to modify and remodel corresponding to certain stimulations, which was considered as the essential mechanism for relearning skills and enhancing an extreme degree of motor function restoration through carefully designed rehabilitative trainings in order to facilitate neural plasticity processes (Lotze et al., 2019; Maier et al., 2019; Michal and Osnat, 2019).

The motor training generally appears to be a complex processes involving multiple stages of movement representations until the certain skill can be executed automatically (Paz et al., 2005; Dayan and Cohen, 2011). Before the consolidation of a motor skill, the skill acquisition typically processes in two main stages: an initial short-term of rapid performance gains followed by a long-term of incremental behavioral improvement at a slower learning pace (Paz et al., 2005). It is believed to ensure the control of behaviors in respond to different motor training stages, and morphological and physiological plasticity may occur at the nervous system under different mechanisms (Dayan and Cohen, 2011). Great efforts have been paid to understand how neuronal circuitry self-modify to meet the changing motor training demands, which indicated the mechanism may be task-, region-, and period-dependent. For example, region-specific plasticity was reported by Yin et al. focusing on the striatum of adult mouse trained on an accelerating rotarod, as neural activity changes in dorsomedial area were found to increase at early stage while the excitability in dorsolateral region was recorded to increase later at a slower-learning stage of training (Yin et al., 2009). Xu et al. measured the synaptic connections in mouse brain, and animal models illustrated task-related rapid formation of synapses can happen in the contralateral motor cortex at the very initial stage of a forelimb reaching training (Xu et al., 2009). Meanwhile, another animal study based upon forelimb activity conducted by Kleim et al. suggested that the induced cortical synaptogenesis and map reorganization more preferentially tended to occur in the late training stage (Kleim et al., 2004). Accordingly, one can be reasonably expected that repetitive training eventually induce significant transmission efficacy changes in synaptic level, as well as the processes of synaptogenesis and cortical map reorganization coordinated by a spectrum of different mechanisms (Plautz et al., 2000). Understanding the modulation of cortical plasticity that self-adapt to the change of training stage would exploit possibilities of training patterns optimization to boost the motor learning or rehabilitation, the importance of which cannot be overstated.

The developments in non-invasive modalities have significantly promoted the flexibility and extensibility of study design to provide internal information of cortical plasticity modulation *in vivo* underlying motor training and skill acquisition. Traditionally, the electroencephalography (EEG) is used to temporally track the spontaneous electrical cortical activities to investigate its adaption to improvements in neural plasticity processes (Kristeva et al., 2007; Omlor et al., 2007). On the contrary, as the brain is an unparalleled delicate organ coordinate human body functioning well whereas energy substrates primarily the oxygen and glucose were required to satisfy neural activities, functional imaging techniques (such as fMRI and PET) have been involved to indirectly investigate the cerebral physiological mechanisms that subsequent to neural metabolism (Landi et al., 2011; Urquhart et al., 2019; Haar and Faisal, 2020; Maes et al., 2020; Matuszewski et al., 2021; Schmidt et al., 2021). The task-specific cortical activation modulation through various motor training paradigms induced was confirmed in a more macroscopic brain level with functional imaging analysis. For example, a fMRI study based upon right-handed isometric force developments revealed distinct training-associated brain activity pattern changes in response to the short- and long-term movement practice, which suggested the mechanisms of plasticity were likely to be different according to behavior stages switched (Floyer-Lea and Matthews, 2005). Ma et al. designed a 4-week training with an explicit finger sequencing task and monitored the behavior-related change in brain through several individual fMRI screening at specific time point, and the fMRI activation map demonstrated the regional activities in primary and supplementary motor area increased initially (pre-training to week 2) and decreased afterward (weeks 2–4) (Ma et al., 2010). Moreover, structural changes in brains were investigated with voxel-based morphometry measurements applied on high-resolution 3D imaging screening. Wenger et al. conducted eight structural MRI scans over a 7-week left-hand writing and drawing practice, and the time-series analysis demonstrated a trend of expansion followed by partial renormalization in gray matter of the primary motor area (Wenger et al., 2016). The discovery further indicated that the experience-dependent cortical modulation induced by motor training was associated not only with the functional but also anatomical changes, and the plastic changes in brain were likely to be mediated differently overtime (Dayan and Cohen, 2011; Wenger et al., 2017; Lövdén et al., 2020). Therefore, better understanding on neural plasticity calls for closer investigations to illustrate more detailed time course of dynamical adaption in brain activity, while most recent studies involved limited discrete measurements partially due to the high expenses, strict environmental requirements, and temporal-resolution limitations using imaging modalities such as fMRI (Ma et al., 2010; Wenger et al., 2016).

Functional near-infrared spectroscopy (fNIRS) is a promising brain-monitoring method using optical techniques to provide neurohemodynamic measurements in the cerebral cortex (Ferrari and Quaresima, 2012). It can non-invasively and dynamically scale the cortical oxygenated and deoxygenated hemoglobin (HbO and HbR) concentration changes which are capable of long-term investigation for clinical applications under various environments (Hatakenaka et al., 2007; Lu and Zhen, 2015; Paol et al., 2018; Chou et al., 2019). In this study, we trained participants with an 8-day right-hand force-control training task for 8 blocks (170 s per block) per day. During the whole training period, the fNIRS was employed to continuously capture the hemodynamic changes subsequent to neural modulation overtime. Another six right-handed untrained participants were also recruited as the control group at the same time. Both the training and control groups were involved to fulfill a similar but new force-control tasks at the 8th day to investigate the learning transfer effects. In addition, transcranial magnetic stimulation (TMS) was used to assess the motor cortical hand representation changes induced by training (Hallett, 2000; Kobayashi and Pascual-Leone, 2003). We expected to record and analyze a time course of consecutive hemodynamic response associated with the experiment-dependent neural activity mediation, which may help better understanding the mechanism of human brain plasticity, addressing problems in practice or intervention, and optimizing treatment in rehabilitations.

## Materials and methods

### Participants

The study was approved and regulated under the Ethics Committee of the Cancer Hospital of Chongqing University. Eighteen right-handed healthy subjects were enrolled in and randomly assigned into the training group and the control group, including 12 (male/female: 6/6, age ranged 22–25 with mean ± standard deviation as 23.08 ± 1.16 years) and 6 (male/female: 4/2, age ranged 22–27 with mean ± standard deviation 23.5 ± 1.87 years) participants, respectively. All subjects had confirmed normal or corrected-to-normal vision, with no history of any physiological or neurological disease. Written informed consent was provided before the experiments. All the participants have ensured themselves not been involved previously in similar or related motor training practice before, and all are guaranteed to conform to the participation criteria to keep normal life activities and avoid any strenuous exercises during the experimental procedure. In addition, questionnaires were taken every day to confirm the physical conditions of participants were kept excellent with sufficient sleep.

### Experimental paradigm

**Figure 1:**
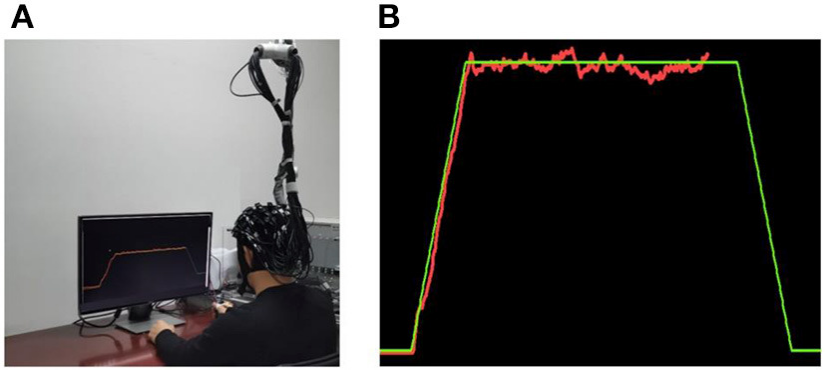
**(A)** Experimental scene. **(B)** The interface of visuomotor force-control task. During the training, the participants were required to product a force (red line) matching the preset force (green line) through visual feedback.

The experiments were allocated to a quiet and dimmed room, and basic setup of a visuomotor force-control designs was shown in Figure 1A. During the force production practices, the subjects were required to sit comfortably with their dominant hands (right hand) holding a dynamometer (PW-2, Pclab, China) which was able to collect the real-time handgrip force signals. Force acquisitions were sampled at 50 Hz and amplified to create an online curve representing the dynamical force outputs during training tasks. A computer screen in front was used to illustrate the preset force-control task (Figure 1B green line) and the force performance of participants (red line) simultaneously, to provide real-time visual feedback for subjects to coordinate the force output. The surface electromyography (sEMG) signals and the cortical hemodynamic changes were measured throughout the whole procedure.

**Figure 2:**
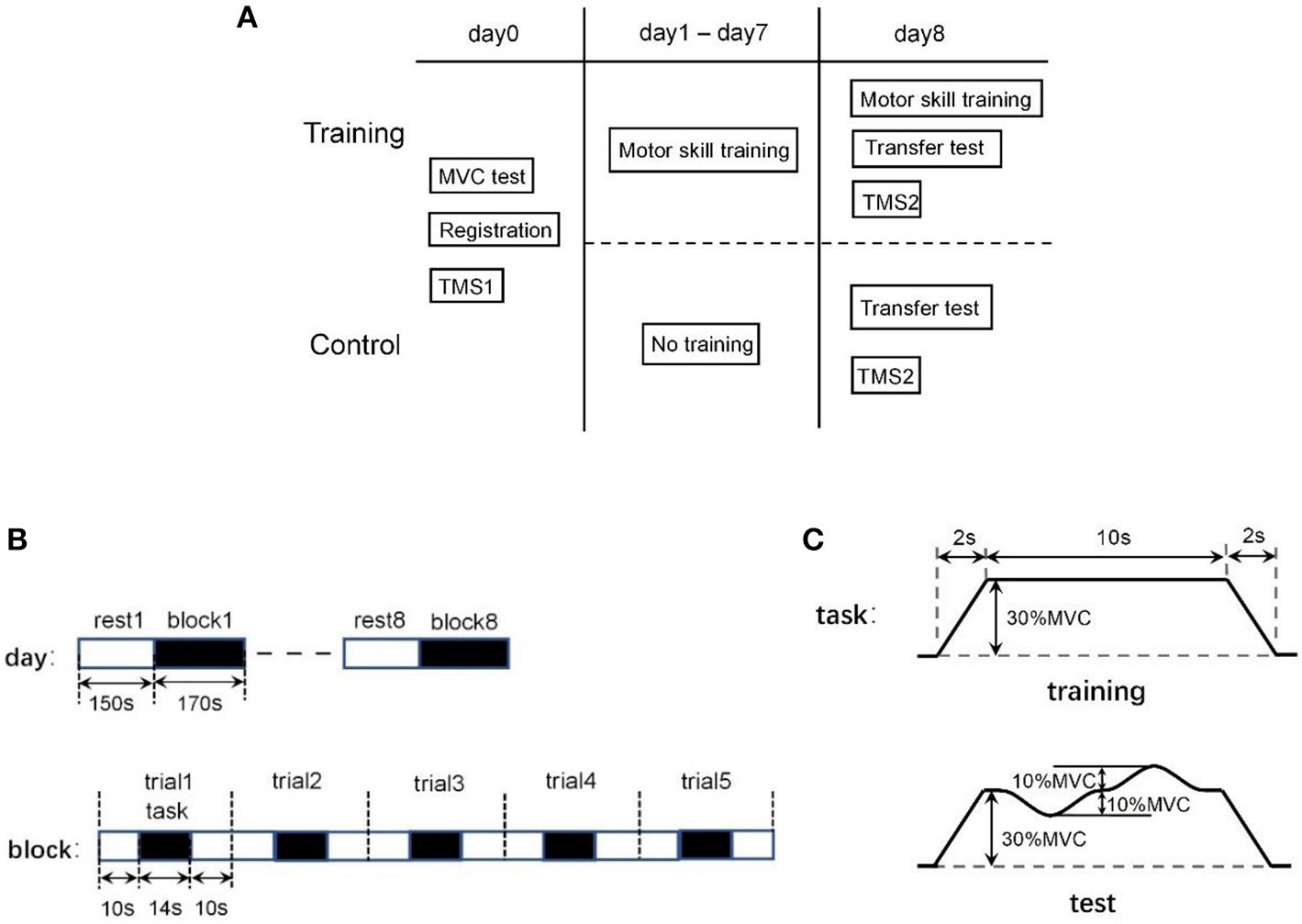
**(A)** Arrangement of experimental paradigm. **(B)** Constitutions of the block-design experiment arrangement: 8 block/day (above) and 5 trail/block (below). **(C)** Each trial includes a force-control training task (above) followed by a 20 s rest to avoid fatigue. In skill transfer experiments, the task was set into a similar but new preset (below).

One day before the protocol onset (referred as Day 0 in the following), all subjects undertook a familiarization visit with registration information documented. The individual MVCs (maximum voluntary contractions) were determined *via* average of three measurements (3 s duration per measurement, separated by at least 3 min rest interval to avoid fatigue). To standardize the difference of force production ability among subjects, the force-control tasks were personalized based upon the certain force proportion (30%) of the individual of participants. Only subjects in the training group were involved in the following 8-day training program, while control group volunteers were assigned to drop by after 8 days without training (Figure 2A).

For training group, all participants underwent a series of training sessions through 8 continuous days. A repetitive block design was utilized, and each block included five trials (Figure 2B). In each trial (34 s), the first was a 10 s preparation period, then 14s force-tracking task was performed followed by a 10 s resting interval to avoid fatigue. The specific handgrip squeezing task (14 s) was preset as steadily increasing the force until 30% MVC in 2 s, then holding with constant output for 10 s, and gradually returning to relax in the next 2 s (Figure 2C). During the task, the participants were asked to squeeze the dynamometer to product a force matching the preset level as much as possible, as the actual output was displayed along with the target force on the screen in front. Each block consists of five force-tracking trials. A training session per day repeated eight blocks with a block interval of 150 s, resulting around 45 min participation per session.

On the last day (referred as Day 8), two additional blocks were assigned to both the training and control group. All subjects were asked to conduct the force-tracking task following a new different preset curve, where the target force was preset to fluctuate (±10% MVC) around 30% MVC (Figure 2C).

### Behavioral data recording and analysis

The force produced while squeezing the dynamometer was collected with a sampling frequency of 50 Hz and normalized with the MVC of individuals. To obtain a quantitative overview of the performance for subjects, a performance score was defined as follows:


(1) $$L_{i}=\{\begin{array}{c} 0,\;|g\left(t_{i}\right)-f(t_{i})|>\delta \\ 1,\;|g\left(t_{i}\right)-f(t_{i})|\leq \delta \end{array}$$

where the *g*(*t*_*i*_) and *f*(*t*_*i*_) represented the normalized data of the actual force production and the preset force level as time functions, respectively. The score *L*_*i*_ at specific time point *t*_*i*_ was defined to earn 1 score if the absolute difference between *g*(*t*_*i*_) and *f*(*t*_*i*_) is equal or smaller than certain expected error δ; otherwise, no score was earned and recorded *as L*_*i*_ = 0. The expected error was set as δ = 0.02.


(2) $$\begin{array}{r} \text{Score}=\frac{100}{N}\sum_{i=1}^{N}L_{i} \end{array}$$

Therefore, the performance score (normalized to 100) can be obtained through the sum of N scores at a sequence of time point as expressed by the equation (2).

Besides, the surface electromyography (sEMG) activities were also collected continuously during the experiment. The extensor carpi radialis (ECR), flexor carpi ulnaris (FCU), and first dorsal interossei (FDI) of the right arm were selected for sEMG recording through electrodes (ZTEMG-1000, ZhiTuo, China). The sEMG signals were then amplified and continuously streamed to the PC at a sampling rate of 1,000 Hz. Offline data analysis was performed in MATLAB 2016a environment (The MathWorks Inc., MA, USA). The 10–450 Hz band-pass filtering and a 50 Hz notch filtering were applied to remove motion artifacts and power frequency interferences. The median frequency (MF) was analyzed in the purpose of monitoring the muscle status free of fatigue through the exercise term.

### Cerebral hemodynamic mapping

**Figure 3:**
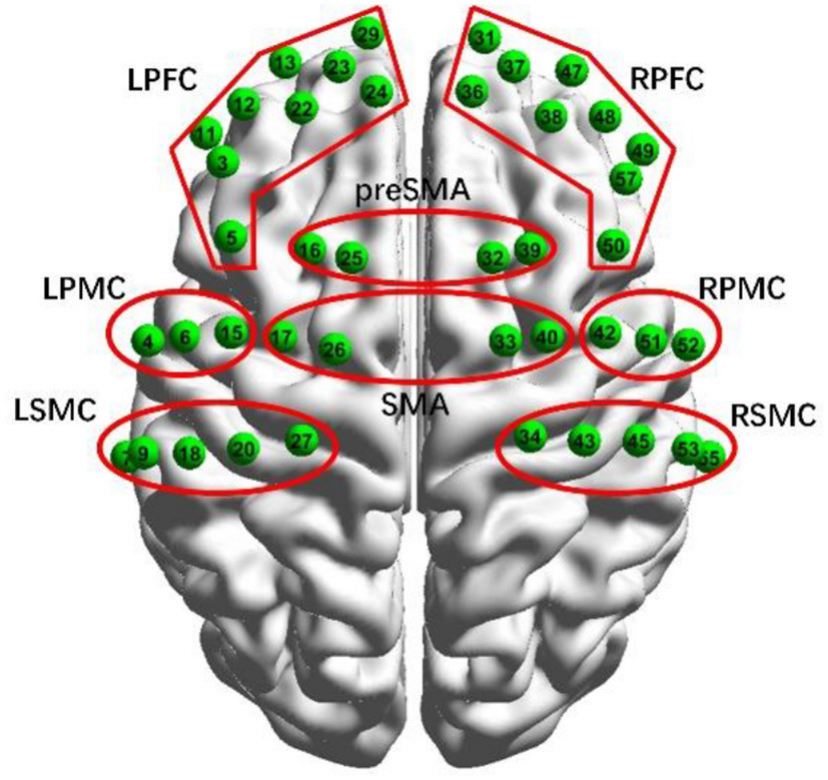
Channel configuration for fNIRS measurement.

The cortical hemodynamic changes were monitored using a dual-wavelength (750 and 808 nm) multi-channel fNIRS system (NirScan, Danyang Huichuang Medical Equipment Co. Ltd., China) which consists of 21 optical emitters and 23 light detectors arranged within the prefrontal cortex and motor areas, with a source-detector distance of 30 mm. The layouts of 42 selected channels formed *via* source-detector pairs distributed on the brain model are illustrated in Figure 3. The configuration of measurement channels covered over the left prefrontal cortex (LPFC), the right prefrontal cortex (RPFC), the left premotor cortex (LPMC), the right premotor cortex (RPMC), the left sensorimotor cortex (LSMC), the right sensorimotor cortex (RSMC), the supplementary motor area (SMA), and the pre-supplementary motor area (pre-SMA), in accordance with the international general 10/20 electrode distribution system (Hatakenaka et al., 2007). The fNIRS measurements were conducted continuously during experiment procedure, with the acquisition sample rate as 17 Hz.

The raw light intensity data were filtered by 0.01–0.2 Hz using five-order Butterworth band-pass filter to remove common physiological noises such as heartbeats and respirations rhythms (Quan et al., 2015), while motion artifacts were also eliminated through sliding window method and PCA algorithm. Therefore, the relative concentration changes of oxygenated hemoglobin were extracted according to the modified Beer–Lambert law. The HbO responses (μM·cm) were then baseline-corrected by the 2 s preparation time window ahead of task execution, and relative cerebral hemodynamics were mapped. The metric of integrated value (IV) of HbO activation which is defined as the integration of active HbO response over task period was involved to quantitatively express the induced hemodynamic changes (Takizawa et al., 2014). Seed-based correlation analysis for functional connectivity (FC) investigation was applied to analyze the interaction between the specific cerebral area and the others. In this study, we specified the day average response of the left sensorimotor cortex (LSMC) as the seed (Lyde et al., 2019; Urquhart et al., 2019), and the Pearson correlation coefficient *r* between the seed and all channels was calculated to evaluate the connection strength. The corresponding topographical maps projecting FC patterns were constructed through BrainNet Viewer (Version 1.7) combined with one-sample *t*-test analysis, where the method was detailed in pervious literature (Xia et al., 2013; Urquhart et al., 2019).

### TMS measurements

The TMS application was used to represent the basic neurophysiological changes of the motor cortex excitability and synaptic plasticity induced by the training. Two TMS measurements were assigned before (Day 0) and after (Day 8) the whole experimental exercise procedure, using a MagPro Compact TMS magnetic stimulator (Medtronic, USA) connected to a figure-eight-shaped coil (Model: C-B60) by a Y-cable. During the measures, the coil was placed tangentially on the scalp with ~45° lateral to the interhemispheric line. The specific magnitude magnetic field of TMS was exerted on brain to evoke a descending impulse potential to generate the motor evoked potential (MEP) on the targeted muscle of first dorsal interossei (FDI) on the right hand, and the excitability parameters were recoded using the Keypoint electromyography-evoked potentiometer. The location of coil was optimized to obtain the MEPs in the FDI and marked on a white swimming cap with the individual nasion–inion and the inter-aural lines recorded as references (Kidgell and Pearce, 2010) to ensure position identity between two TMS measurements pre- and post-experiment procedure. The rest motor threshold (RMT) was referred as the minimum magnetic stimulation intensity of the target muscle that induces >50 μV MEP at least 5 times in 10 consecutive stimulations at rest. The magnetic stimulus intensity needed to evoke the MEP of target muscle closest to 1 mV was defined as SI1mV, and the intensities of TMS stimuli delivered to subjects were expressed in the form as a percentage of SI1mV. Fifteen MEPs of peak-to-peak amplitude each were recorded with 70, 80, 90, 100, 110, and 120% of SI1mV, and the first 10 effective MEPs were used to calculate a mean MEP for each stimulus intensity. Therefore, the input–output curves (IO curves) were established with corresponding slopes of IO curve calculated to represent the change of stimulus intensity-dependent recruitment within cortex projection to hand muscle during the training.

### Statistical analysis

The statistical analyses were performed. For behavior investigations, the one-way repeated-measurement ANOVA was used to test the performance changes across training days with *post-hoc* analysis conducted using pairwise comparisons with Bonferroni corrections; meanwhile, the behavioral performance in the transfer-test post-training was tested using two-sample *t*-test, comparing differences between the training and control groups. The two-sample *t*-test was used to test the differences of induced hemodynamic activation between the training and control groups, and the comparisons of hemodynamic activation across regions were examined by two-way repeated-measurement ANOVA. A paired *t*-test was also used to evaluate the difference between the excitability parameters of two assigned TMS tests. The significant level was set as *p* < 0.05.

## Results

### Behavioral performance

**Figure 4:**
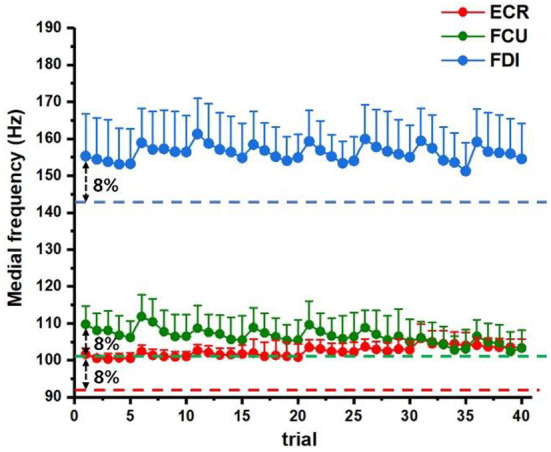
The sEMG signals were used for fatigue status monitoring. The averaged MF statistics of 8 days were summarized above for target muscles (ECR: red, FCU: green, and FDI: blue) during training. The dashed lines correspond to the preset fatigue threshold (8% decline level of the averaged MF of first trial according to previous references).

All participants were confirmed to have sufficient rest to keep in good state during the whole training procedure, and according to the sleep questionnaires, the sleep durations for participants are 7.7 ± 0.3 h. The median frequency (MF) of sEMG signals was calculated to monitor the muscle status of fatigue through training. Figure 4 illustrates the averaged MF stream of muscle (ECR, FCU, and FDI) trial by trial (5 trial/block × 8 block/day = 40 trial/day, numbered as trial #1-#40 on x-axis) of 8 days. A typical threshold of muscle fatigue was set as 8% decline level of MF averages of the initial trial (dashed line) according to several previous studies. The MF analysis of sEMG ensured all participants were kept in good condition during the exercise procedure.

**Figure 5:**
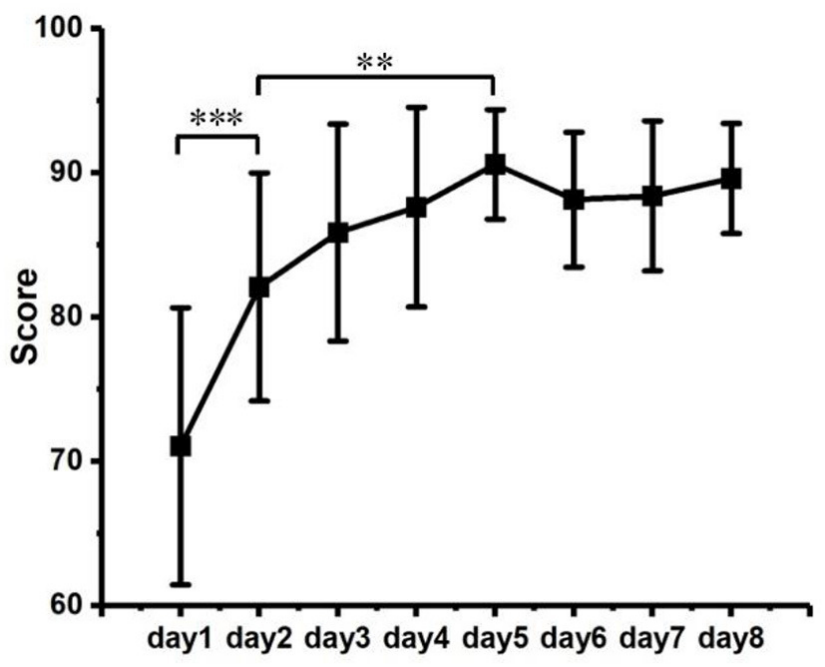
Scores of behavior performance across training duration. Significant level: ** as *p* < 0.01 and *** as *p* < 0.001.

The behavioral performance was quantified by the scores defined in Section Behavioral data recording and analysis directly relative to how precise the participants produce force according to preset level, and Figure 5 summarizes the average daily performance scores of all participants, which significantly improved from initial 71.0 ± 10.00 (mean ± standard deviation) to 89.60 ± 3.82 (*p* < 0.001). The results expressed relative improvements in accuracy of force production induced by repeated practice, with an increasing trend that ascend fast in the first a few days and then slow down to reach a plateau after training. According to repeated-measurement ANOVA, a fast improvement generally raised within the first 2 days (*p* < 0.001); then, the performance continued to increase progressively with a slower pace which still lead to a significant increment in average score from day 2 to day 5 (*p* = 0.008) and tended to be stabilized after 5 days of training (*p* > 0.05). Besides, though the tasks were normalized by individual MVCs, unlike the initial fast improvements, the period lengths for participants to reach their performance score plateau were not in accordance with each other, which indicated the individual differences still existed in learning speed.

### Cerebral hemodynamic results

**Figure 6:**
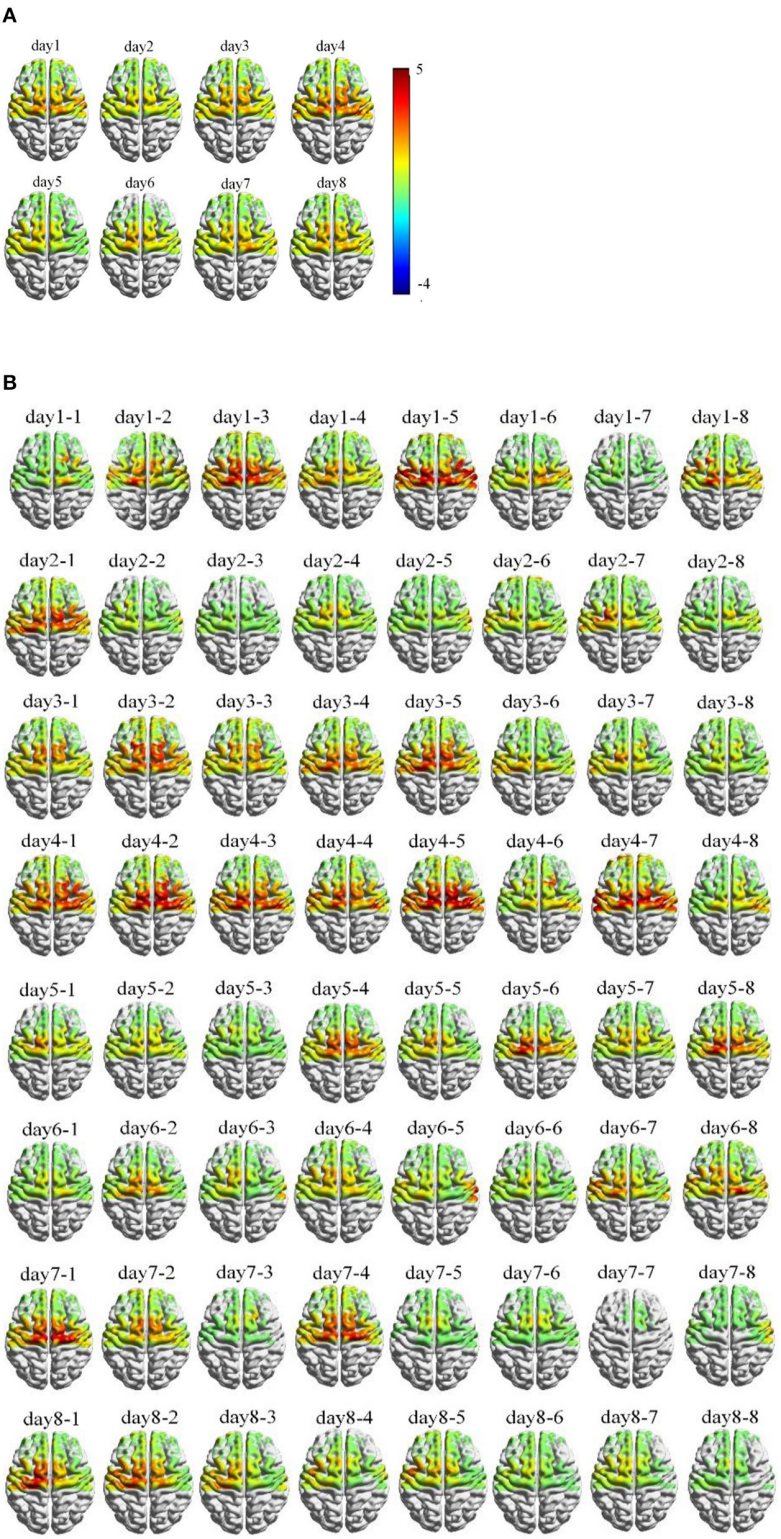
Example of cortical hemodynamic response of one typical participant during training process, mapped by Δ[HbO] activation and arranged in temporal sequence **(A)** per day; **(B)** per block.

A typical example of averaged cerebral HbO activation atlas during the training procedure was illustrated in Figure 6, sequenced by day (Figure 6A) and by block (Figure 6B), respectively. Generally, a trend of cerebral hemodynamics can be observed as “expansion to renormalization,” which referred a visible increase of neural activation induced by the early training followed by a gradual decrease response in later tasks. Similar hemodynamical change was also found in other participants but under different learning rates. The time length that different participants spent to reach their cerebral activation peak varied from 3 days to 7 days, averaged as 4.33 ± 1.07 days.

**Figure 7:**
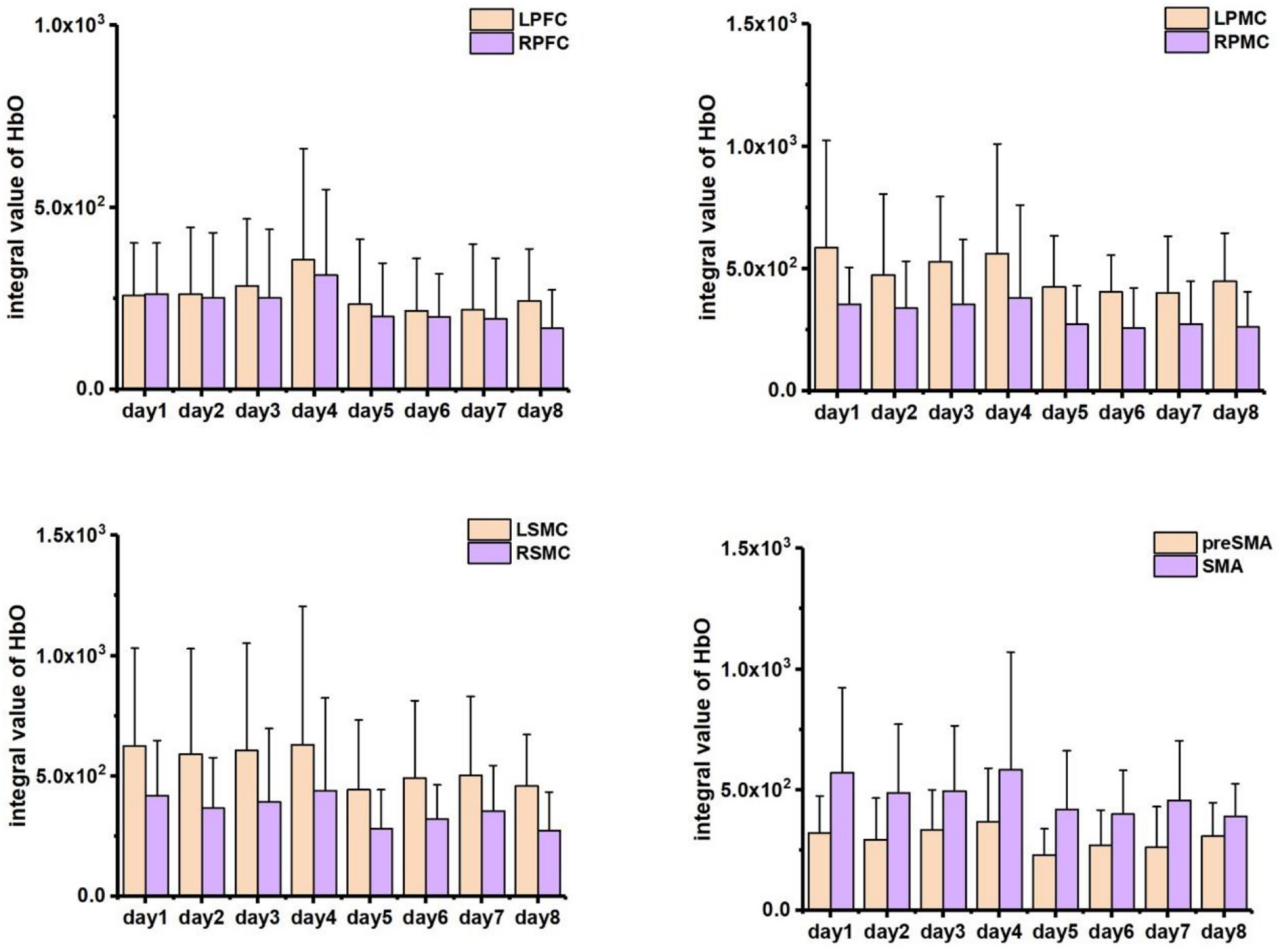
Averaged integral value of Δ[HbO] activation within ROIs during experiments.

**Figure 8:**
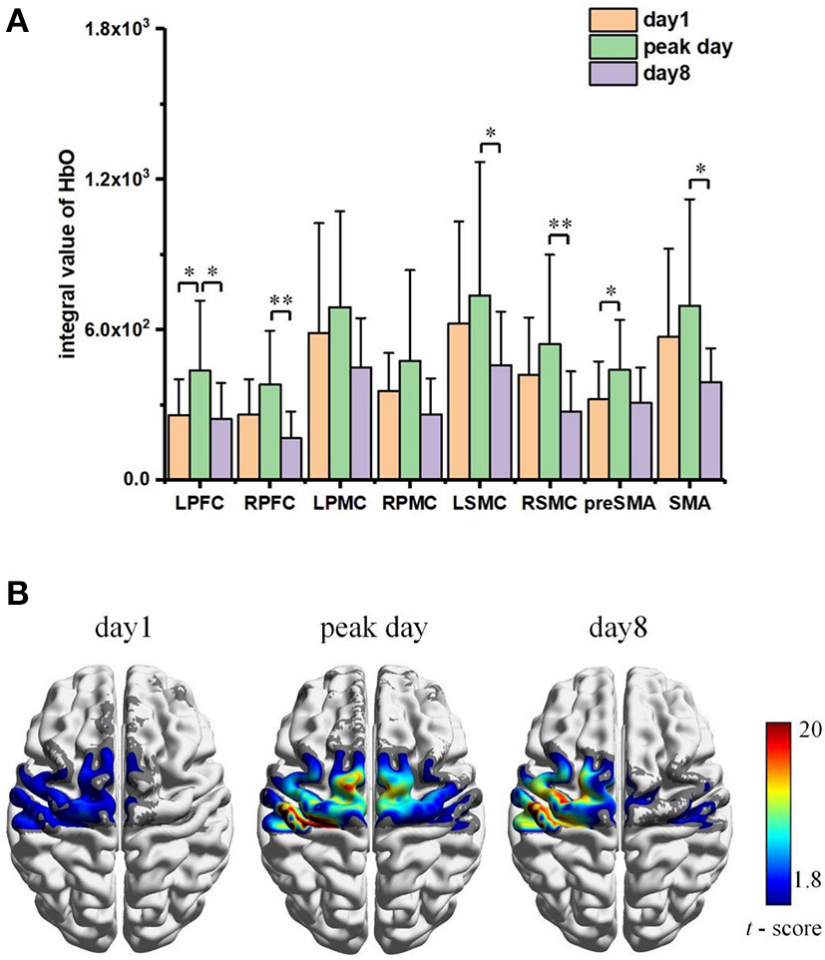
**(A)** Averaged Integral Value of Δ[HbO] activation within ROI specific to day1, peak day, and day 8. Significant level: * as *p* < 0.05 and ** as *p* < 0.01. **(B)** Corresponding FC pattern maps, with only statistically significant FC strength are shown (*p* < 0.05).

A statistical summary of IV data relative to different interested brain regions was included in Figure 7. Higher activation was found in the left side of PMC (significance: *p* < 0.05) and SMC (without significance) in accordance with contralateral control rule in the brain as all the participants confirmed right-handed. However, the SMA region and part of pre-SMA area exhibited bilateral activations during early training and tended to shift to left lateral activation in the later experiments. Along with the experiment procedure, the HbO response to the task decreased with time; thus, the cerebral activation in first half course of training (days 1–4) was visibly higher than that of the last half course (days 5–8). No significant changes (*p* > 0.05) were found according to the daily averaged data, one possible reason probably due to the individual differences on learning progress. To reduce the influence of learning variation, we collected the data specific in the initial day (day 1), the day when participants reached their peak activations according to the grand mean HbO response of all channels (peak day) and the last day of training (day 8), and then summarized the relative fNIRS response in Figure 8A. The activation increase can be observed within all areas on peak day comparing to initial, with significant changes found within the pre-SMA (*p* = 0.030) and LPFC (*p* = 0.034), while significant decrease (*p* < 0.05) was shown from peak day to the last day for the area of L/RPFC (*p* = 0.022 and 0.004), L/RSMC (*p* = 0.027 and 0.008) and SMA (*p* = 0.032). There were no significant differences (*p* > 0.05) between the activation of days 1 and 8 for the area of LPFC (*p* = 0.752), L/RPMC (*p* = 0.273 and 0.113), LSMC (*p* = 0.076), SMA (*p* = 0.104), and pre-SMA (*p* = 0.820).

The functional connectivity pattern atlas with the seed at LSMC during the specific days (Day 1, Day of peak activation, and Day 8) was mapped in Figure 8B. Only the regions with statistically significant FC strength (*p* < 0.05) were shown. According to the FC pattern map, training-induced statistically significant FC strength appeared along with the practice progress, with an expansion of active region first (Day 1 vs. Peak Day) followed by a re-contraction (Day 8 vs. Peak Day). The participants showed significant LSMC-seed FC strength primarily to the region of internal-LSMC, SMA, and LPMC at peak day. The active regions for significant FC were pretty similar comparing Day 1–Day 8, the FC strength was increased against the initial status which indicated an effect of repeated practice; while comparing to peak day, the functional connectivity on day 8 remained high active within the left side of above regions (LSMC, SMA, and LPMC), the strength of connection in some channels which corresponds to the right side of LSMC and SMA regions got reduced or even diminished, however.

### Skill transfer performance post-training

**Figure 9:**
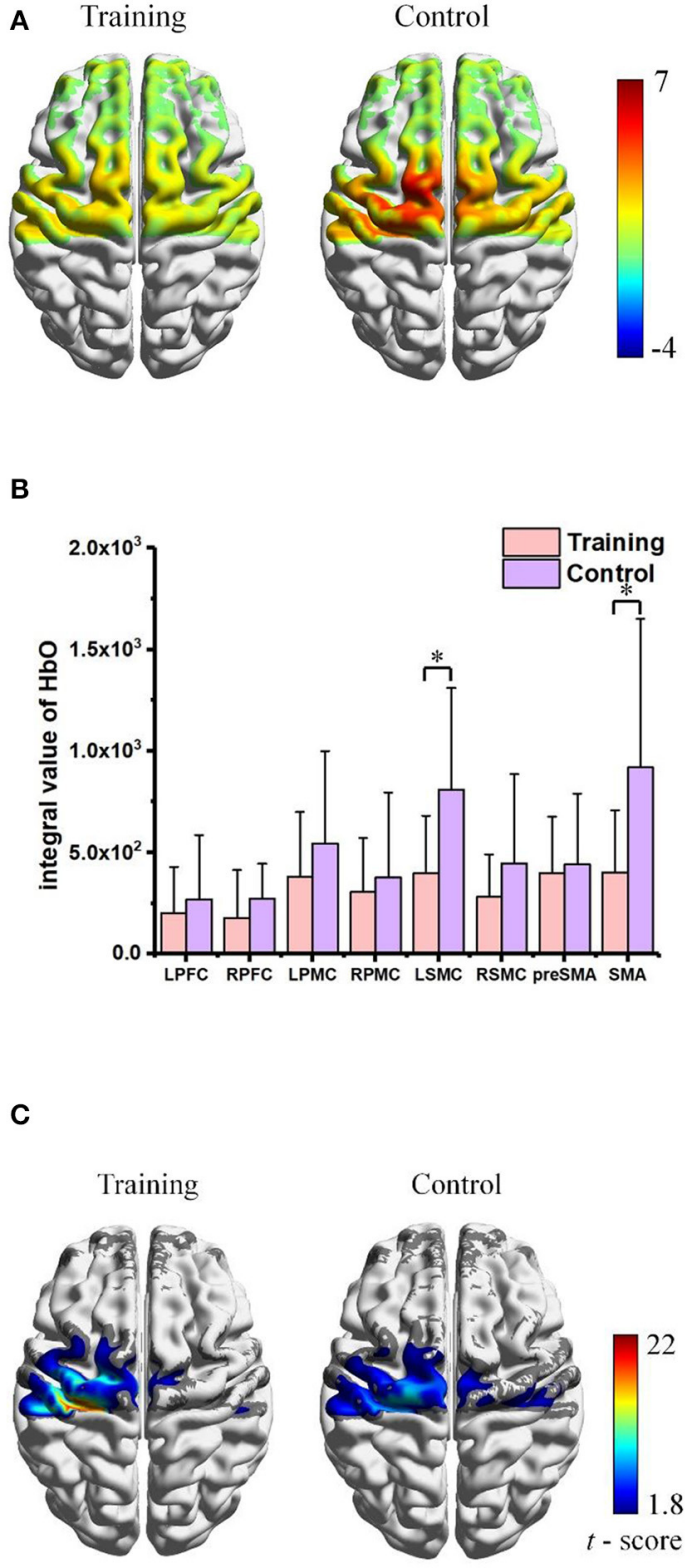
Skill transfer experiments post-training: **(A)** Comparison of cortical hemodynamic activation maps. **(B)** Averaged Integral Value of Δ[HbO] activation within ROIs. Significant level: * as *p* < 0.05. **(C)** Corresponding FC pattern maps, with only statistically significant FC strength are shown (*p* < 0.05).

The transfer of learning was investigated through a related force production task experiments following a new unfamiliar preset. The participants of training group demonstrated a significant better performance compared to the controls (*p* < 0.001), as the corresponding performance scores were 75.32 ± 10.61 vs. 49.83 ± 7.19, respectively. The distribution of averaged cerebral hemodynamics response induced by the transfer-test tasks is mapped in Figure 9A, and numerical statistics of regional HbO changes are summarized in Figure 9B. Expected fNIRS response increasements were observed in the control group, indicating a higher activation for the untrained participants to fulfill the same tasks. Significant increase (*p* < 0.05) was found in the left sensorimotor cortex (LSMC) and the supplementary motor area (SMA) which were supposed to be the regions for motor control. In addition, the functional connectivity patterns were also analyzed. As Figure 9C illustrates, the trained participants represented statistically significant and stronger FC strength within LSMC region which is pretty similar to the functional connectivity pattern induced by training task of Day 8, while the untrained participants represented a FC pattern more closely to that induced by the initial training.

### Training-induced changes on MEPs and IO curves

**Figure 10:**
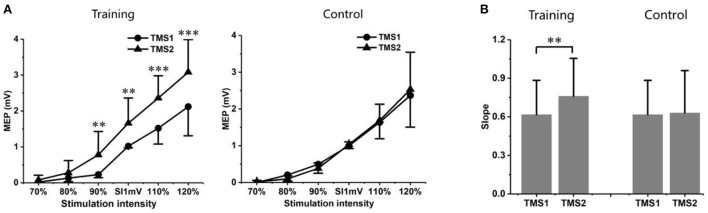
**(A)** IO curves measured MEPs in the FDI muscle as a function of magnetic stimulation intensity before (Day 0: TMS1, marked as solid circle •) and after (Day 8: TMS2, marked as solid triangle ▴) motor practice for training (left) and control (right) groups, respectively. Significant MEPs differences were found between TMS1 and TMS2 measurements within the training group, while no statistical differences were found between the two MEPs measurements in control group. **(B)** The slopes of the IO curve were obtained within the approximately linear part (90–120% of SI1mV). The statistical differences were also found within the training group, indicating the effect on the descending motor pathway caused by training. Significant level: **p < 0.01 and ***p < 0.001.

Two TMS assignments were applied to investigate the neurophysiological changes of participants in training and control group. Figure 10 shows the IO curves measured on the right FDI muscle before (Day 0: TMS1) and after (Day 8: TMS2) the experiments, illustrating the relationship of MEPs (mean ± standard deviation) against intensity of magnetic coil stimulation delivered to the motor cortex. The MEPs on target muscle increased significantly according to the same stimulation intensities (*p* < 0.05 for stimulation intensity: 90%, 100%, 110%, and 120% of SI1mV) after the training procedure (Day 8 vs. Day 0) for the training group, while no significant differences were found for the untrained participants in control group. The slope of the IO curves (Figure 10B) was also calculated within the approximately linear part (90–120% of SI1mV) and compared above the curves on Figure 10, which was able to give a simplified description revealing the functional change of response to stimulation according to the descending motor pathways. The slopes were significantly increased after the motor practices for the trained participants (*p* < 0.05).

## Discussion

This study aimed to explore the hemodynamic modulation of cortical plasticity induced by motor training. We conducted an 8-day block-design motor training experiment with multiple neural and physiological parameters recorded continuously. This practice involved repeated visuomotor force-control tasks which asked the participants to produce preset certain level of hand-grab force as precise as possible through visual feedback. Therefore, this study employed fNIRS to monitor the response of cortex area within the prefrontal cortex and motor areas (L/RPFC, L/RPMC, L/RSMC, SMA, and pre-SMA) during the experiments. Temporal evolutions of cortical hemodynamic activation mapped by fNIRS were monitored throughout the whole experiment procedure. The hemodynamic activation and FC patterns analysis along with the force-control performance data suggested a neural activity change and cortical map reorganization as the experience cumulated while training. The before- and after-training TMS measurements of both the training and control groups further expressed the cortical excitability change induced by long-term training practice.

The repetitive practice generally involved a motor skill acquisition process until long-term retention (Willingham, 1998; Dayan and Cohen, 2011). Though the durations were highly specific to tasks, previous studies have demonstrated the learning phase can be characterized into stages which were typically initialized by an early fast learning stage followed by a later slow learning stage, wherein improvements in performance were continuously developed but under relatively fast and slow incremental rates across training (Ungerleider et al., 2002). In our study, the behavior performance was quantified by a score metric representing the accuracy of execution. The performance scores clearly illustrated the temporal improvements along with training process, which increased rapidly during the first day of practice, then slowed down but continuously obtain further gains until relative plateau consolidated. Therefore, the behavior performance results of this study were in agreements with the learning stages. Furthermore, intermittent task design was used to avoid physical fatigue induced by the muscle contractions, with the sEMG monitoring to ensure the effect of muscle fatigue was eliminated.

The temporal evolution of hemodynamic activation tracked the morphological changes of neurophysiological modulations induced by training. Unsurprisingly, the cortical hemodynamic activation mapped by fNIRS showed a left-tended excitability in the area of PMC and SMC which followed the contralateral control rule for right-handed participants. In the meantime, expected bilateral activations in SMA and pre-SMA were observed in early training stage. This activation pattern can be attributed to that the visuomotor force-control task involved several small hand muscles (such as the muscles of dorsal interossei) and the right-arm muscles (such as ECR and FCU) working together to accomplish the complex and fine adaptions to keep certain accuracy of force production at the level of 30% individual MVC, which is in accordance with pilot findings that the secondary cortical motor areas may tend to contralateral-activated for simple movements but bilateral-activated for advanced functions in voluntary actions (Derosière et al., 2014; Iso et al., 2016). Though behavior performance increased steadily as the training was repeated, the hemodynamic response to an execution with higher accuracy was still reduced, which indicated the establishment and stabilization for the individualized and optimized neural representations as experience cumulated in practices (Makino et al., 2016; Wenger et al., 2017; Lövdén et al., 2020). The activation area of SMA and pre-SMA regions was observed visibly shrink and diminished to left lateral, which is in accordance with the previous theory that as resources in secondary motor areas can be minimized as movements performed with higher level of automaticity after skill acquisition (Poldrack et al., 2005; Puttemans, 2005; Steele and Penhune, 2010). Animal models in pilot findings also revealed the trends of initial bilateral-activation followed by contralateral-activation after learning (Paz et al., 2005). Similar results were also reported for human subjects studies, for example, Xia et al. conducted an investigation based upon upper limb training, and similar hemodynamic changes were found in the sensorimotor cortex (SMC), supplementary motor area (SMA), and premotor cortex (PMC) of both patients with stroke and healthy participants during different experimental conditions (Xia et al., 2022).

The learning-related brain changes during repetitive practices and skill acquisition were a perpetual process rooted in accounts of plasticity mechanism (Dayan and Cohen, 2011). A variety of published studies had reported task-relevant activation increases along with morphological brain expansions induced by lasting training (Debaere et al., 2004; van der Graaf et al., 2004; Floyer-Lea and Matthews, 2005; Lehericy et al., 2005). However, as early researchers recognized that the constraints must exist to achieve an ultimate representation for continuous increasing in brain, indeed a few studies addressed observed decreases in learning experiments (Molina-Luna et al., 2008; Quallo et al., 2009; Ma et al., 2010; Wenger et al., 2016). Taking into account pilot research on both human and animal models, Wenger et al. put forward the expansion–renormalization model which was prominent theoretical for plastic changes in humans (Wenger et al., 2017). The model predicted the dynamical neural changes induced by the learning processes followed a sequence of expansion, selection, and renormalization, which could result in an initial increased response caused by cortical synaptogenesis and structural enhancements while the cortical activation may complete or partially renormalized to the baseline due to the selection of efficient circuit. The selective formation/elimination of synaptic junction (Xu et al., 2009) and neural reorganization (Kleim et al., 2004) in the process of task training had been reported by a few animal models. In this study based on human participants, the temporal evolution of cortical hemodynamic activation analysis followed the expansion–renormalization model in the means of both the amplitudes and the active regions.

Functional connectivity analysis further investigated the brain changes. The FC patterns of our results also exhibited the characteristics of expansion–renormalization trends for the region areas with statistically significant FC strength. However, the FC strengths (LSMC seed-based) within those regions were increased, which indicated that the connectivity pattern enhancement by training still remained when cortical activation returned to the baseline. Further proofs were found through the skill transfer experiments and TMS measurements. Comparing the participants with and without training, the trained subjects showed significant performance enhancement when facing a similar task than those untrained. According to the learning transfer theory, transfer may occur at a subconscious level when encountering problems sufficiently similar to the original learned if the skill execution has achieved automaticity (Lövdén et al., 2010). Therefore, the performance of the trained participants could be enhanced by the skill transfer, since all of them had already achieved a performance plateau through the 8 days of repeated practices. Last but not the least, the TMS results demonstrated significant increase for the trained participants after fulfilling the whole experiments. As TMS delivered magnetic stimulations to motor area and recorded the evoked motor action potentials (MEPs) on the target muscle, the results of changes on MEPs confirmed that a neural plastic change, which can be referred relative to the long-term potentiation (LTP) in plasticity mechanism, had been induced by training which facilitated functional connections of the descending motor pathways (Rioult-Pedotti et al., 1998; Rosenkranz et al., 2007a,b).

Nevertheless, our study also has certain limitations. First, the recruited sample size was small which may affect the statistical power of study. Considering the existence of individual differences, expanded studies with more subjects are needed to validate the cerebral hemodynamics relative to characteristics of brain plasticity during motor learning. Further factors such as the impact of subjects' age may also be involved in near future schedules. Second, the TMS technique was used for verifying the inducing of neural excitability changes pre- and post-training, wherein the individual SI1mV was determined initially before training participation and kept used as constant inputs of stimulation for MEP measurements post-training without renormalization. The results may indicate the tendency of MEPs facilitation caused by motor practices according to the amplitude differences comparing the training vs. control group; however, considering the temporal variation, further experiments with strict stimulation intensity normalization on identified muscles should be taken for quantitative comparison across days. Third, the cortical response to motor learning was expected to be task-related and sensitive to the challenges encountered according to task difficulties, as optimized task difficulty and practice sequence assignments were considered to accelerate the learning procedures through the mechanism for quantifying the relationship between task difficulties and learning benefits remained unestablished. Therefore, it would be important to extend the training protocols with different tasks under various kinematic parameters and different difficulty levels in future work to explore the advanced cerebral task- and difficulty-related adjustments in motor learning mechanisms. In addition, all participants involved in this study were confirmed with healthy conditions, which limited the direct analytical implementations for clinical rehabilitation strategies due to the pathological changes in patients with motor function disorders. To extend investigations for clinical effects in rehabilitation conditions, further research that includes motor dysfunctional populations would be of great significance in the following studies since the motor function recovery somehow can be considered as re-learning procedures for lost skills.

## Conclusion

This study monitored the cortical hemodynamic modulation during a continuous 8-day visuomotor force-control designed training. The temporal evolution of cortical activation mapped by fNIRS showed a dynamic neural response following the expansion–renormalization trends. The functional connectivity patterns indicated an increase of neural connections still maintained when the hemodynamic response returned to the initial status after motor skill acquirement. The skill transfer experiments then confirmed a learning transfer enhancement on behavior performance with new similar tasks for the trained participants. Finally, the TMS measurements further proved a neural facilitation on the descending motor pathway induced by the long-term training. The current findings of this study tried to explore the sequence of cortical changes under the plasticity mechanism, which may contribute to a fundamental knowledge that assists the optimization on physical exercises and rehabilitation treatments.

## Data availability statement

The raw data supporting the conclusions of this article will be made available by the authors, without undue reservation.

## Ethics statement

The studies involving human participants were reviewed and approved by the Ethics Committee of the Cancer Hospital of Chongqing University. The patients/participants provided their written informed consent to participate in this study.

## Author contributions

YW designed the experiment, collected and analyzed the data, and drafted the manuscript. SF designed the experiment, collected and analyzed the data, interpreted of the data, and drafted the manuscript. RY collected and analyzed the data. XW participated in experiment setup and interpreted the results partly. LC and WH proposed and supervised the project, and revised the manuscript. All authors approved the final manuscript.

## Funding

This work was supported by the National Natural Science Foundation of China (31800824), the National Key R&D Program of China (2020YFC2004200), and the Chongqing Science & Technology Program (cstc2018jcyjAX0390).

## Conflict of interest

The authors declare that the research was conducted in the absence of any commercial or financial relationships that could be construed as a potential conflict of interest.

## Publisher's note

All claims expressed in this article are solely those of the authors and do not necessarily represent those of their affiliated organizations, or those of the publisher, the editors and the reviewers. Any product that may be evaluated in this article, or claim that may be made by its manufacturer, is not guaranteed or endorsed by the publisher.
